# The Shifting Flow: Aortic Root Pseudoaneurysm with Dynamic Multichamber Fistulization after Aortic Valve Surgery

**DOI:** 10.1016/j.case.2025.06.009

**Published:** 2025-08-15

**Authors:** Sneha Limaye, Ryan O'Connor, Eric Czinn, Jean-Pierre Iskandar, Farhan Bajwa

**Affiliations:** Division of Cardiology, University of Rochester, Rochester, New York

**Keywords:** Aorta aortic valve, Echocardiography hemodynamics valve repair

## Abstract

• ARPA with multidirectional fistulae is rare.
• Complex ARPAs with LA and LV fistulae are rare complications of AV surgery.
• Hemodynamic interactions of these fistulae may resemble multivalvular physiology.

## Graphical abstract

**Figure:**
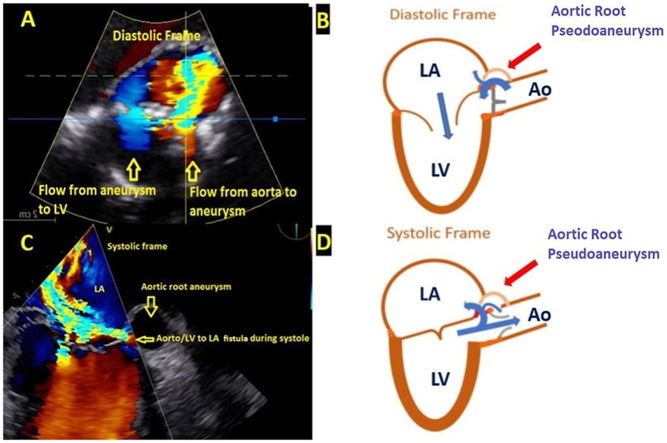

## Introduction

Aortic root pseudoaneurysms (ARPAs) are rare echocardiographic findings. Multidirectional intracardiac fistulae are uncommon complications of ARPA. We present a case of an ARPA with fistulization to the left ventricle (LV) and left atrium (LA), with dynamic shifts in fistula flow direction depending on the phase of the cardiac cycle.

## Case Presentation

**Figure 1:**
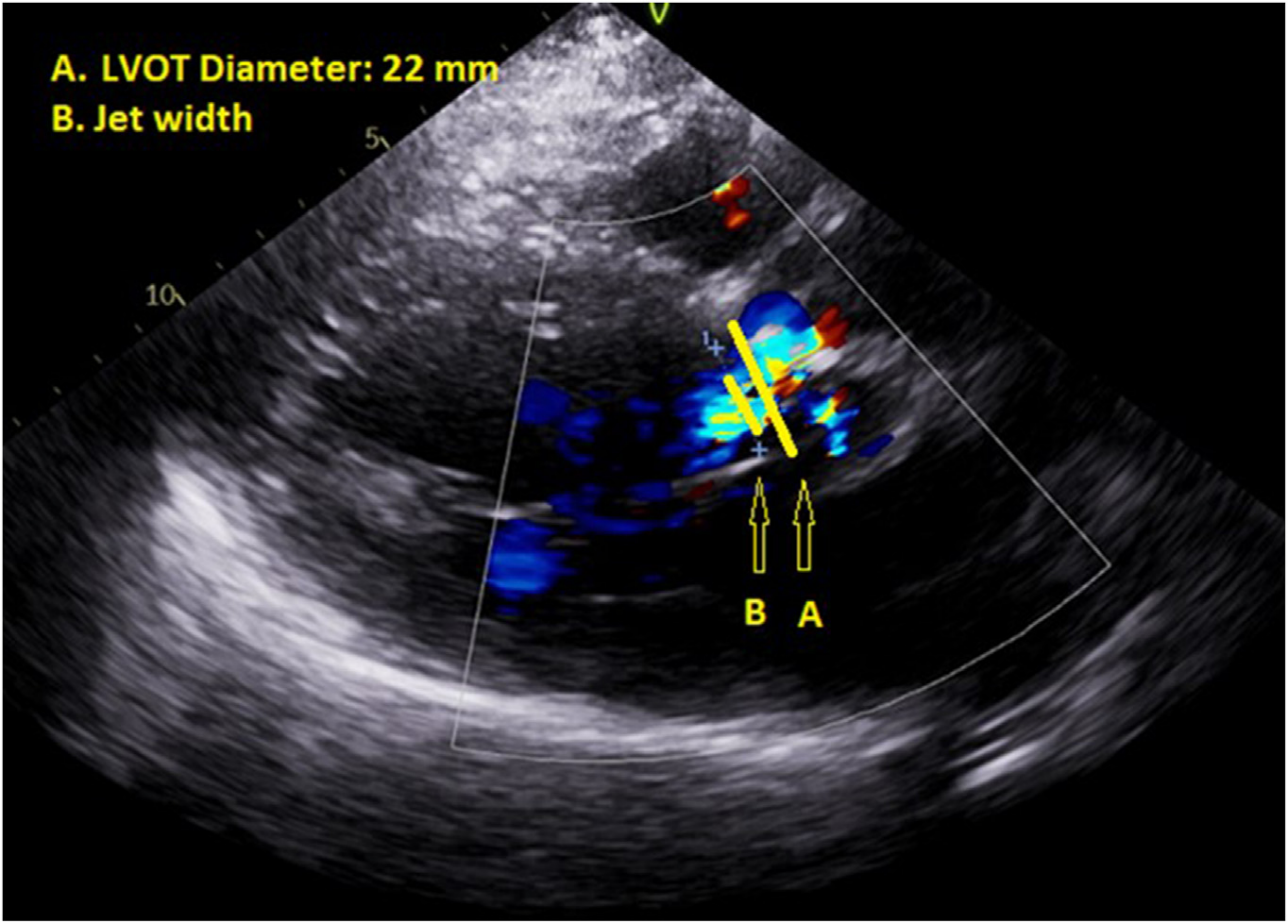
Two-dimensional TTE, parasternal long-axis diastolic view with color-flow Doppler, demonstrates a dilated left ventricular cavity, increased left ventricular wall thickness, and a bioprosthetic AV with moderate paravalvular regurgitation (jet width [B] to LVOT [A] ratio of 40%). *LVOT*, Left ventricular outflow tract.

A 73-year-old patient with a history of bicuspid aortic valve (AV), bacterial endocarditis treated with tissue AV replacement (AVR) with a bioprosthetic valve and mitral valve repair, atrial fibrillation treated with multiple cardioversions, and hypertension presented with subacute exertional dyspnea and palpitations. The patient's multivalvular surgery was performed 6 years ago. They denied experiencing fevers or chills and exhibited no focal infectious symptoms. Blood pressure was 160/60 mm Hg with a heart rate of 64 beats per minute. Exam was significant for a grade 2/4 diastolic murmur. They were also due for a routine transthoracic echocardiogram (TTE) for bioprosthetic valve surveillance. The patient described mild functional limitation and reported exertional fatigue, consistent with New York Heart Association functional class II status. They also reported intermittent palpitations. The TTE showed a bioprosthetic AV with significant paravalvular regurgitation assessed qualitatively (jet width ratio of 40%; Figure 1, Video 1). The patient's previous TTE performed 1.5 years prior showed less significant paravalvular aortic regurgitation (AR).

**Figure 2:**
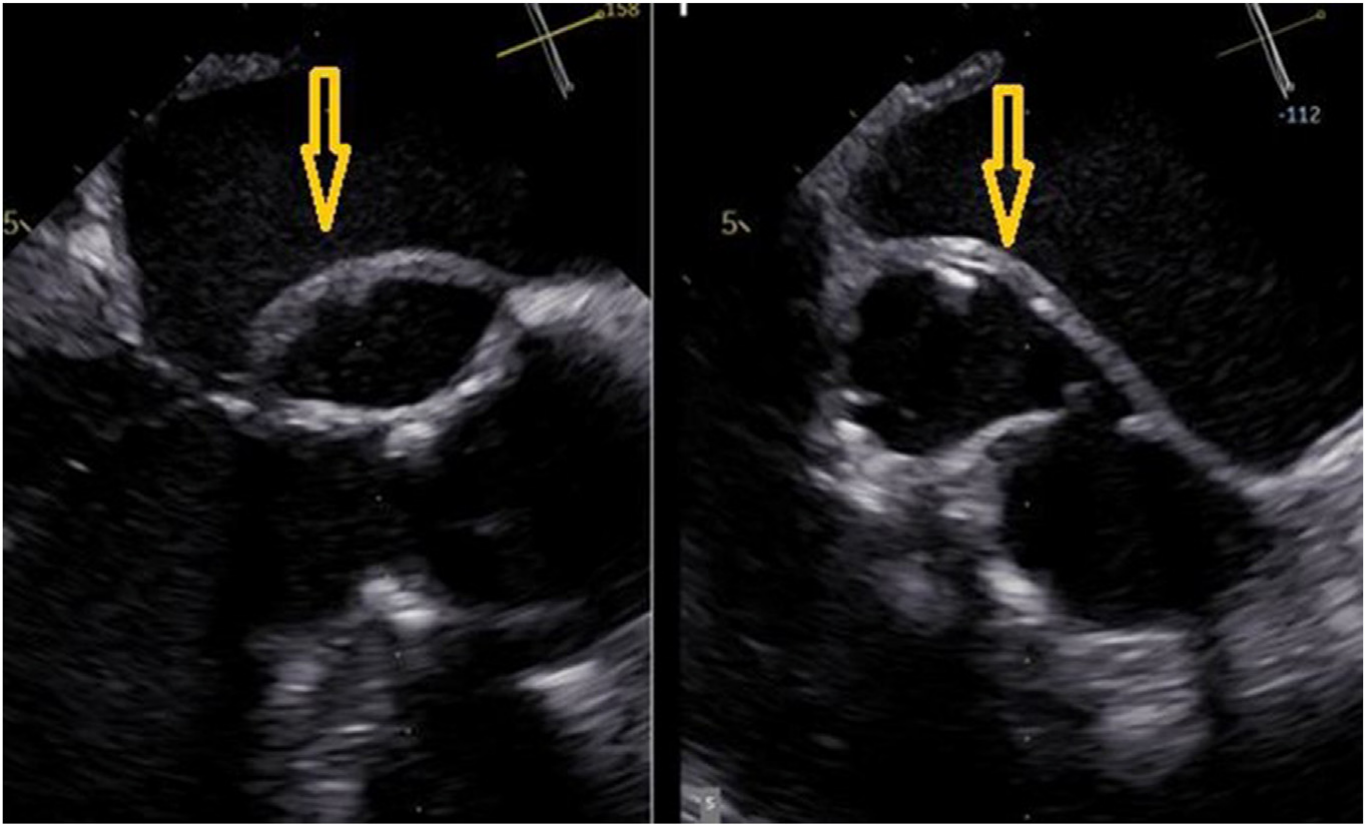
Two-dimensional TEE, mid-esophageal window, simultaneous orthogonal zoomed biplane systolic display, demonstrates the AVR and a large ARPA with systolic expansion (*arrows*).

**Figure 3:**
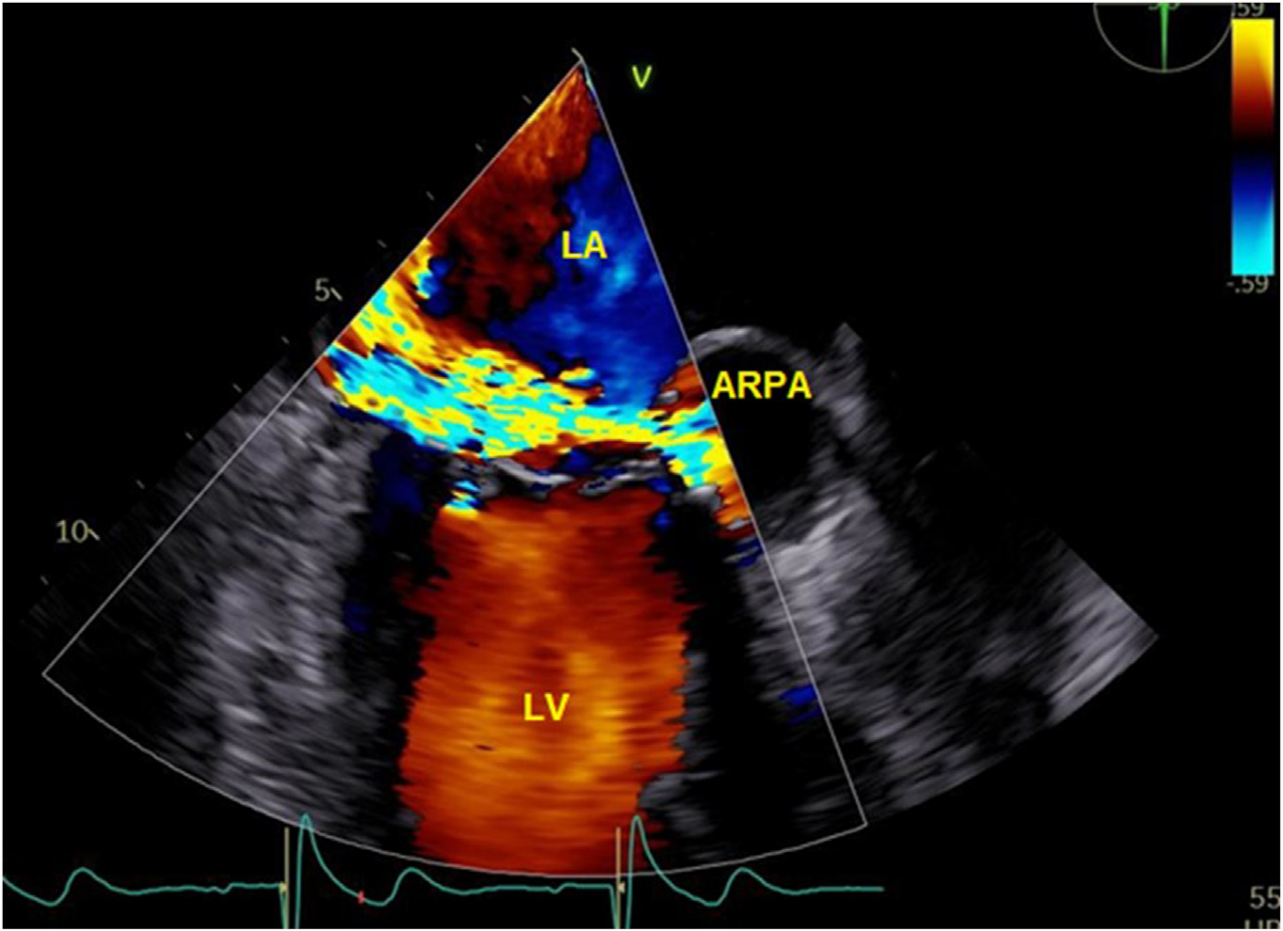
Two-dimensional TEE, mid-esophageal window, long-axis (90°) zoomed systolic display with color-flow Doppler, demonstrates the large ARPA with fistulous communication with the LA (eccentric, posteriorly directed).

**Figure 4:**
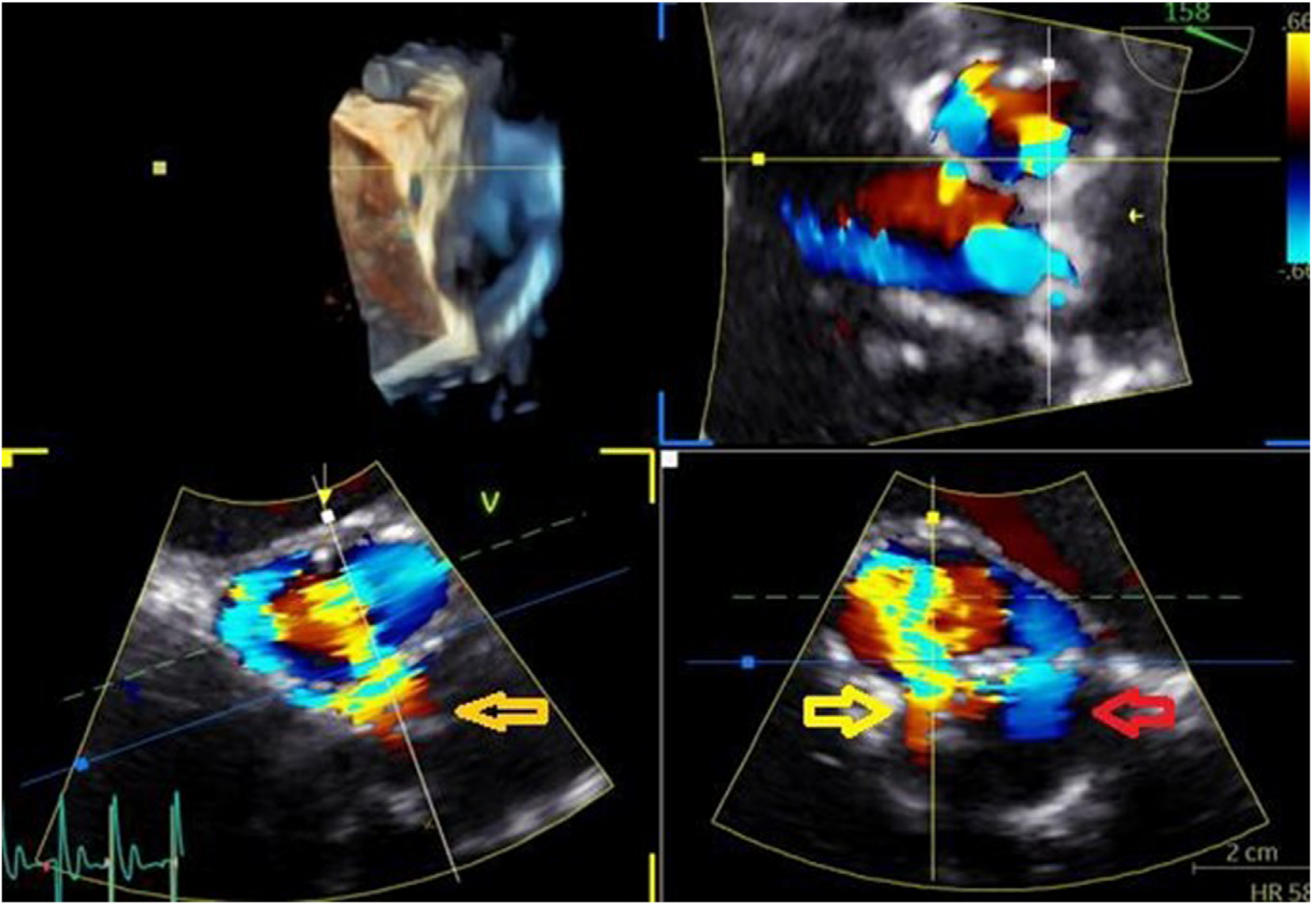
Three-dimensional TEE, mid-esophageal window, postprocessing multiplanar reconstruction simultaneous orthogonal zoomed systolic displays with color-flow Doppler, demonstrates the ARPA and its communication with the aorta (*yellow arrow*) and the LV (*red arrow*).

A transesophageal echocardiogram (TEE) revealed AR and an ARPA involving the left sinus of Valsalva, measuring 4.5 × 2.6 cm (Figure 2, Video 2). The ARPA communicated with the aorta, LV, and LA (Figures 3 and 4, Videos 3-6).

**Figure 5:**
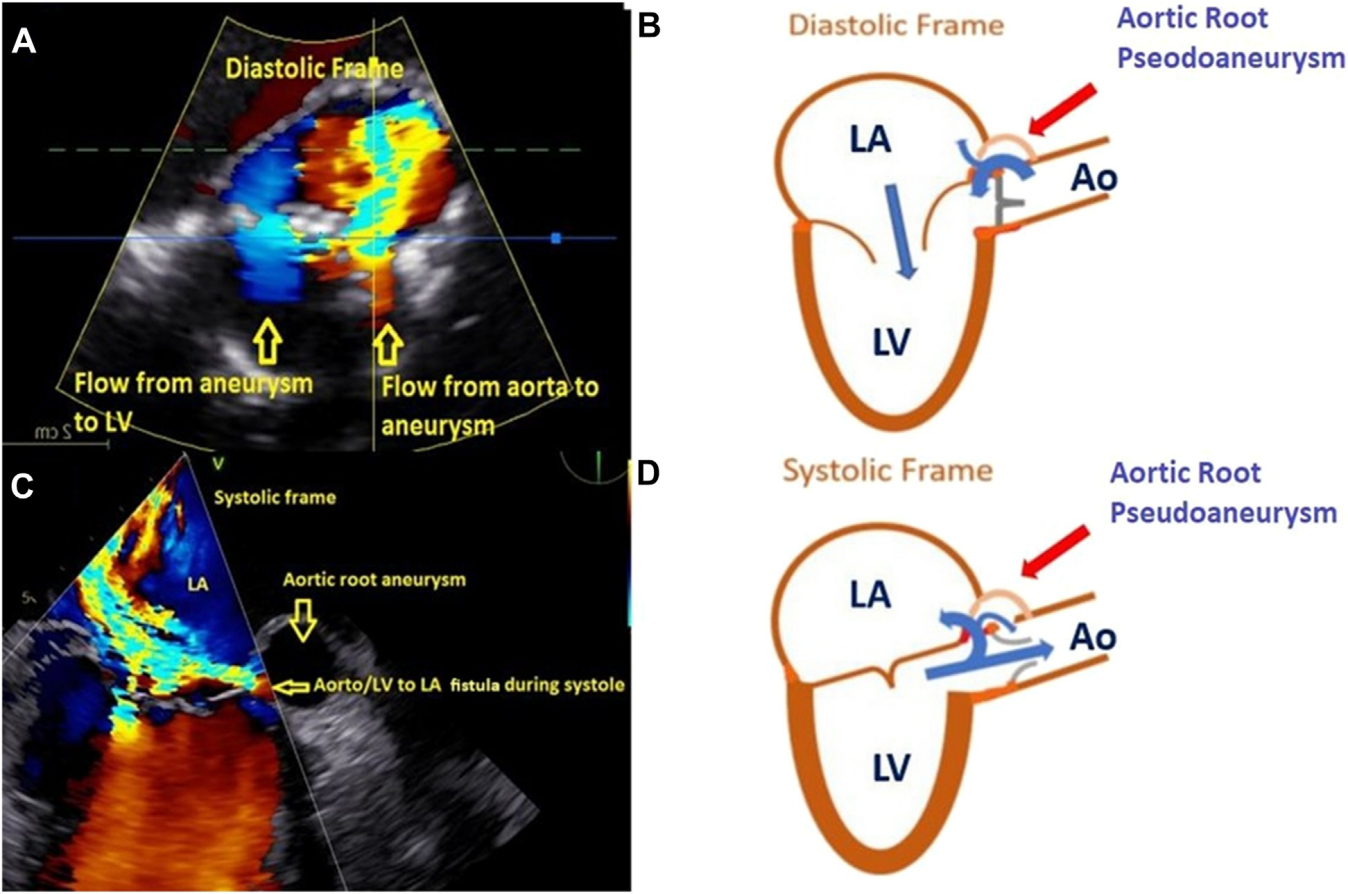
Central illustration of the ARPA arising from the left sinus of Valsalva, with fistulous connections to the LV and LA. Two-dimensional TEE with color-flow Doppler in diastole (**A**; mimics AR) and systole (**C**; mimics MR) with comparison drawings (**B** and **D**), demonstrates the ARPA with fistulous communication to the Ao, LV, and LA. *Ao*, Aorta.

**Figure 6:**
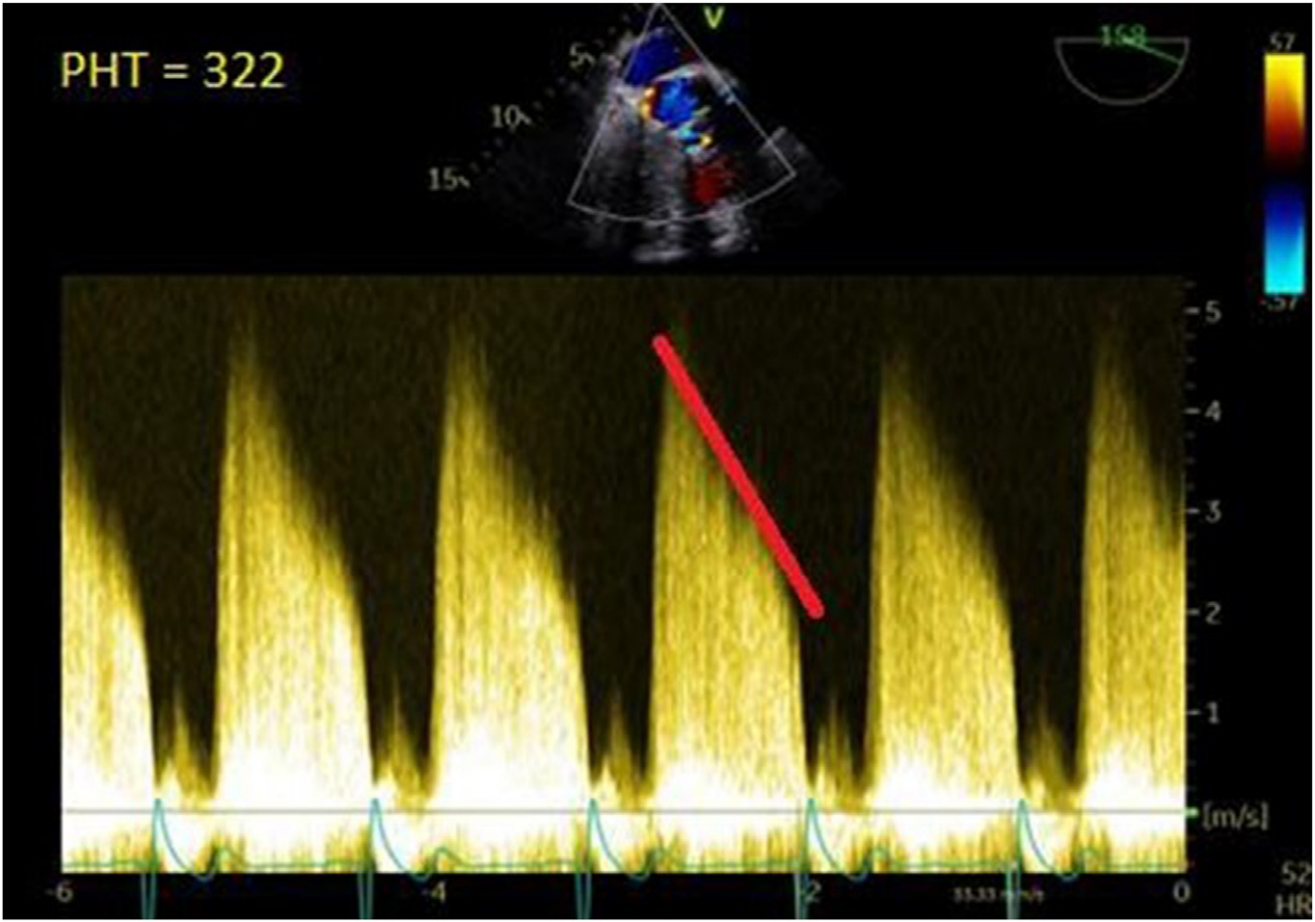
Two-dimensional TEE, mid-esophageal window, long-axis (158°) view with color-flow Doppler-guided continuous-wave Doppler, demonstrates a dense spectral Doppler profile of AR with a short pressure half-time of 322 ms (*red line*).

During systole, the ARPA-LA fistula exhibited characteristics similar to an LV-LA fistula, while during diastole, it resembled an aorto-LA fistula (Figure 5). Flow was observed from the LV into the ARPA during systole and subsequently into the LA. The pseudoaneurysm functioned as a conduit between the LA and LV, with flow appearing as an eccentric regurgitation jet into the LA, mimicking severe mitral regurgitation (MR). Systolic flow reversal was noted in the pulmonary veins, resembling severe MR physiology. During diastole, flow entered the ARPA from the aorta, with most of it directed toward the LV, while a small amount appeared in the LA. Holodiastolic aortic flow reversal was present in the descending thoracic and abdominal aorta, resembling severe AR. The pressure half-time of the jet was 322 ms (Figure 6). Physiology similar to MR and AR contributed to left ventricular volume overload.

To accurately evaluate left ventricular remodeling in response to this complex physiology, TEE was combined with TTE using an ultrasound-enhancing agent. Significant left ventricular dilation was identified, with a left ventricular end-diastolic volume index of 141 mL/m^2^. This dilation was attributed to the ARPA's fistulous connections with the aorta, LV, and LA, resulting in substantial left ventricular volume overload. The patient remained afebrile, and multiple blood cultures showed no growth.

Because of multiple complex fistulae, which produced physiology akin to severe MR and AR alongside marked left ventricular dilation, the patient underwent redo sternotomy with AVR and aortic root repair, achieving excellent recovery. Intraoperative exploration confirmed 40% valve dehiscence at the left ventricular outflow tract with partial aortoventricular discontinuity with pseudoaneurysm and a resultant LV-LA fistula. All cultures from the explanted valve were negative, and the patient was discharged in stable condition. A follow-up TTE performed at 15 weeks confirmed a well-seated bioprosthetic AV with no evidence of valvular or paravalvular regurgitation (Videos 7 and 8).

## Discussion

Aortic root pseudoaneurysms are an uncommon but serious complication following AV surgery, typically arising from degenerative changes, prior infections, or surgical dehiscence. Although data on ARPA are scarcer than for congenital sinus of Valsalva aneurysms, one study estimates that pseudoaneurysms occur in 5% to 10% of patients after aortic root procedures, with a high rupture risk if untreated.^1^ They most often develop at the aortic annulus or near surgical anastomoses and may progressively enlarge over time.^2^

**Figure 7:**
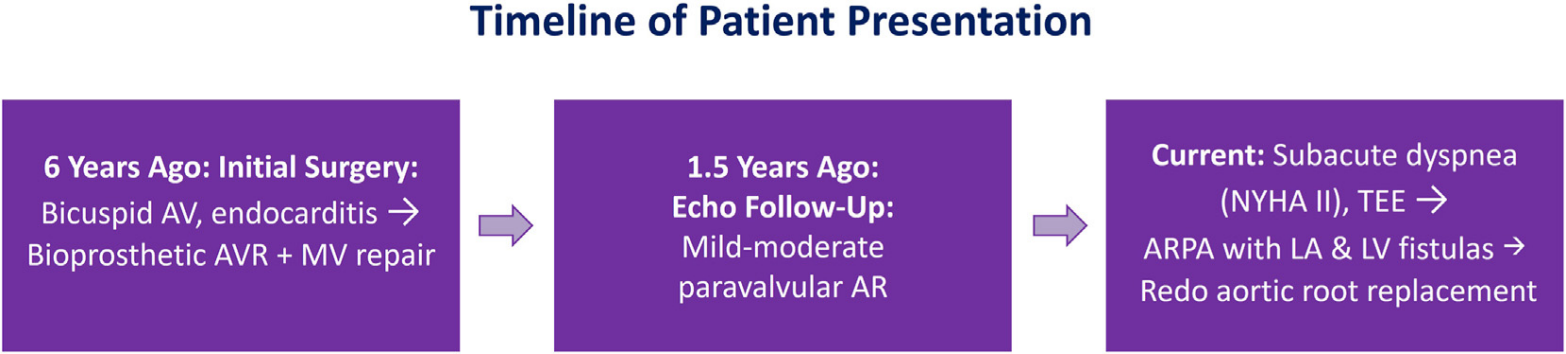
A condensed timeline highlights key clinical milestones from the initial bioprosthetic valve surgery to the current presentation and planned reintervention.

The natural history of ARPA progressing to intracardiac fistula formation remains poorly characterized, with no established guidelines for management. However, case reports indicate considerable rupture rates, causing symptoms such as dyspnea, fatigue, and chest pain and occasionally leading to severe hemodynamic compromise.^3^ Fistulization involving the left sinus and LA appears to be exceedingly rare, being documented in only 1 of 149 cases in one report.^4^ Published case series suggest that onset of symptoms can occur months to years after the original aortic root surgery.^5^ Additionally, valve dehiscence linked to ARPA formation has been documented, underscoring the need for prompt intervention to avert catastrophic outcomes.^6^ This timeline closely reflects our patient's presentation, which manifested several years after the initial operation (Figure 7).

A complex aorto-LV-LA fistula is extraordinarily unusual. In this case, the combination of ARPA, bioprosthetic valve failure, and multiple fistulous connections created systolic flow from the LV to LA (mimicking MR) and diastolic flow from the aorta to LV (mimicking AR). This 2-phase shunting led to left ventricular diastolic dysfunction, marked left ventricular dilation, and left ventricular systolic dysfunction. The resultant effects on coronary and systemic perfusion were vital considerations.

Although both MR and AR components raise left ventricular preload, AR also heightens total left ventricular afterload (wall stress and workload).^7^ In this patient, the pressure half-time of the prosthetic AR jet measured 322 ms, which is classified as moderate, but must be interpreted alongside the superimposed MR physiology that elevates left ventricular diastolic pressures. The tridirectional shunt between the aorta, LV, and LA effectively replicated a multivalvular lesion, prompting a clinical approach akin to that used in mixed valvular disease. The differential diagnosis for the mechanism of paravalvular bioprosthetic valve regurgitation included structural and degenerative changes, recurrent infectious endocarditis, and premature bioprosthetic valve failure.

Definitive treatment in this setting required careful assessment of the ARPA's dimensions, the number and size of fistulous tracts, shunt volume, and degree of left ventricular remodeling.

## Conclusion

The presence of an ARPA with multiple intracardiac fistulae represents a significant complication following AV surgery, likely due to degenerative changes in the absence of active infective endocarditis. The hemodynamic and physiological effects of these multidirectional intracardiac fistulae closely mimic multivalvular disease, highlighting the need for thorough evaluation when considering surgical intervention. Clinical vigilance is essential in patients with a history of surgical AVR, and the use of high-quality imaging modalities is crucial for accurately assessing both the anatomical and physiological impact of the condition.

## Ethics Statement

The authors declare that the work described has been carried out in accordance with the following guidelines: This is a retrospective single-patient case report compiled exclusively from fully deidentified clinical data contained in the electronic medical record. No prospective intervention, experiment, or animal study was performed; all care the patient received was standard, clinically indicated treatment delivered in the usual course of practice. Under 45 CFR 46.104(d)(4) and corresponding institutional policy, such retrospective, deidentified chart reviews are exempt from further ethical review.

## Consent Statement

The authors declare that since this was a noninterventional, retrospective, observational study utilizing de-identified data, informed consent was not required from the patient under an IRB exemption status.

## Funding

The authors declare that funding for this report was provided by a Continuing Medical Education (CME) Fund allocated for attending physicians at our institution.

## Disclosure Statement

The authors reported no actual or potential conflicts of interest relative to this document.

## Supplementary Data

Video 1 Two-dimensional TTE, parasternal long-axis view with color-flow Doppler, demonstrates a dilated left ventricular cavity, increased left ventricular wall thickness, normal global left ventricular systolic function, and a bioprosthetic AV with moderate paravalvular regurgitation.

Video 2 Two-dimensional TEE, mid-esophageal window, simultaneous orthogonal zoomed biplane display, demonstrates the AVR and a large ARPA with systolic expansion.

Video 3 Two-dimensional TEE, mid-esophageal window, long-axis (90°) zoomed display with color-flow Doppler, demonstrates the large ARPA with fistulous communications with the aorta (red/yellow color flow) and the LV (blue color flow).

Video 4 Two-dimensional TEE, mid-esophageal window, short-axis (45°) display with color-flow Doppler, demonstrates the large ARPA with fistulous communications with the aorta (2 o'clock position) and the LV (11 o'clock position).

Video 5 Two-dimensional TEE, mid-esophageal window, long-axis (90°) display with color-flow Doppler, demonstrates the large ARPA with fistulous communication with the LA (eccentric, posteriorly directed).

Video 6 Three-dimensional TEE, mid-esophageal window, postprocessing multiplanar reconstruction simultaneous orthogonal zoomed systolic displays with color-flow Doppler, demonstrates the ARPA and its communication with the aorta and the LV.

Video 7 Two-dimensional TTE, apical 3-chamber view obtained at follow-up visit, demonstrates normal global left ventricular systolic function, postoperative septal motion, and the well-seated bioprosthetic AV without findings of an ARPA.

Video 8 Two-dimensional TTE, apical 3-chamber view with color-flow Doppler obtained at follow-up visit, demonstrates normal appearing AVR without evidence of valvular or paravalvular regurgitation.
